# Misinterpreting Diarrhea-Predominant Irritable Bowel Syndrome and Functional Diarrhea: Pathophysiological Highlights

**DOI:** 10.3390/jcm12185787

**Published:** 2023-09-05

**Authors:** Giusi Desirè Sciumè, Ginevra Berti, Christian Lambiase, Italia Paglianiti, Vincenzo Villanacci, Francesco Rettura, Antonio Grosso, Angelo Ricchiuti, Nicola de Bortoli, Paolo Usai Satta, Gabrio Bassotti, Massimo Bellini

**Affiliations:** 1 Gastrointestinal Unit, Department of Translational Sciences and New Technologies in Medicine and Surgery, University of Pisa, 56010 Pisa, Italychristian.lambiase93@gmail.com (C.L.);; 2 Nuclear Medicine Unit, Department of New Technologies and Translational Research in Medicine and Surgery, University of Pisa, 56010 Pisa, Italy; 3 Institute of Pathology ASST-Spedali Civili, University of Brescia, 25121 Brescia, Italy; 4 Gastrointestinal Unit, ‘P. Brotzu’ Hospital, 09121 Cagliari, Italy; 5 Gastroenterology & Hepatology Section, Department of Medicine, University of Perugia Medical School, 06129 Perugia, Italy

**Keywords:** irritable bowel syndrome, microscopic colitis, bile acid malabsorption, diarrhea, BAM, IBS, functional disorder

## Abstract

Irritable bowel syndrome with predominant diarrhea (IBS-D) and functional diarrhea (FD) are disorders of gut–brain interaction characterized by recurring symptoms which have a serious impact on the patient’s quality of life. Their pathophysiology is far from being completely understood. In IBS-D growing evidence suggests that bile acid malabsorption (BAM) could be present in up to 30% of patients. Microscopic colitis (MC) is a well-known cause of watery diarrhea and some patients, at first, can be diagnosed as IBS-D or FD. Both BAM and MC are often responsible for the lack of response to conventional treatments in patients labelled as “refractory”. Moreover, because BAM and MC are not mutually exclusive, and can be found in the same patient, they should always be considered in the diagnostic workout when a specific treatment for BAM or MC is unsatisfactory. In the present review the possible shared pathogenetic mechanisms between BAM and MC are discussed highlighting how MC can induce a secondary BAM. Moreover, a brief overview of the current literature regarding the prevalence of their association is provided.

## 1. Introduction

Irritable bowel syndrome with diarrhea (IBS-D) and functional diarrhea (FD) are the two major disorders of gut–brain interaction characterized by watery diarrhea [1], with a worldwide prevalence of 1.2% and 4.7%, respectively [2]. These two conditions, defined and diagnosed by the Rome IV criteria [3], represent an important health issue due to their high social costs, morbidity and possible association with other conditions, such as other disorders of gut–brain interactions or psychological disorders (e.g., anxiety and depression) [2,4,5,6]. The diagnostic workout of chronic diarrhea often may appear uncertain, especially in the presence of refractory symptoms. Guidelines focus their attention on the importance of making a positive diagnosis of these disorders, but in patients not responding to conventional treatment (e.g., lifestyle changes, nutritional approaches and pharmacological therapies [1,7]), in the absence of alarm symptoms, organic diseases must be ruled out [1].

Some studies have estimated that up to one third of patients diagnosed as IBS-D or FD may actually have a bile acid diarrhea (BAD), and that about 1% of the general population suffers from bile acid malabsorption (BAM) [8,9]. Furthermore, microscopic colitis (MC), a condition clinically characterized by watery diarrhea, can be found in IBS-D or FD suspected patients. MC can be classified, according to histopathological findings, in two predominant forms [10]:

- Lymphocytic Colitis (LC), in the presence of an increased number of intraepithelial lymphocytes (≥20 per 100 surface epithelial cells) combined with an increased inflammatory infiltrate in the lamina propria and a not significantly thickened collagenous band (<10 mm).
- Collagenous Colitis (CC), in the presence of a thickened subepithelial collagenous band (≥10 mm) combined with an increased inflammatory infiltrate in the lamina propria.

When the abovementioned histopathological diagnostic criteria are not fully matched, it is also possible to identify an incomplete MC [10].

The overall prevalence of MC in the general population is 119/100,000 [11] and this entity accounts for 13% of patients with chronic watery diarrhea. Interestingly, a recent meta-analysis showed a prevalence of MC of 9.8% in IBS-D patients [12].

In patients meeting the criteria for IBS-D or FD not responding to conventional therapy, all recent guidelines suggest that BAM should be ruled out through a positive diagnosis with a SeHCAT (75-selenium homocholic acid taurine) test [13]. When SeHCAT is not available, a serum 7α-hydroxy-4 cholesten-3-one (C4) assessment [14] or a serum fibroblast growth factor 19 (FGF19) [14] or a dosage of 24-h fecal output of fecal bile acids may be performed [15]. If these tests are not available, at least an empirical trial with cholestyramine should be carried out [1].

The SeHCAT is a nuclear medicine test. Due to its high sensitivity and specificity, it is considered the gold standard among all tests for diagnosing BAM [16]. It is simple, fast, and well tolerated by patients. It requires two scans one week apart. It measures the whole-body retention of a radiolabeled taurine-conjugated bile acid analogue (^75^Se) after seven days; a retention value of ≤10–15% is usually considered diagnostic of BAM [16].

In refractory patients it is also mandatory to perform a colonoscopy with random biopsies of the entire colon to rule out MC [12,17,18,19].

It should also be taken into account that BAM and MC may be associated, because BAD has been reported to be present both in collagenous colitis (CC) (41%) and lymphocytic colitis (LC) (29%) [12,17]. Likewise, a SeHCAT test should be considered in MC patients unresponsive to conventional therapy with budesonide (e.g., 9 mg daily for 6 or 8 weeks) [10].

The aims of our narrative review are to elucidate the importance of the association of MC and BAM in patients with chronic watery diarrhea (IBS-D or FD) and to explore the possible shared pathophysiological mechanisms between these two conditions, on the basis of the most recent literature.

## 2. Methods

In this narrative review we included studies regarding the presence of chronic watery diarrhea in human adults. A comprehensive online search without temporal restriction of PubMed (MEDLINE), Scopus, and the Science Citation Index was made using the following terms and operators “Microscopic Colitis AND Bile Acid Malabsorption”. We included only studies published in English and manual cross-referencing was performed.

We found 29 papers dealing with the coexistence of BAM and MC in patients with chronic watery diarrhea. We included in this narrative review 13 clinical studies and 2 case reports regarding the coexistence of BAM and MC (Table 1).

## 3. Bile Acid Diarrhea and Microscopic Colitis in the Current Literature

### 3.1. Clinical Studies on BAM and MC

Clinical studies included in our review considered patients with chronic watery diarrhea as predominant symptom. In two studies [17,27] patients reported abdominal pain associated with chronic diarrhea, although the authors did not classify these patients as IBS-D, according to currently available Rome criteria. One of them [17] reported no significant differences regarding the symptoms’ response to therapy (including abdominal pain) between MC patients with or without BAM.

Regarding the diagnostic tests for BAM, high heterogeneity was reported. Most studies diagnosed BAM with the SeHCAT test, but the cutoff for BAs retention on day 7 varied between studies: in most cases BAM was diagnosed if the SeHCAT retention was <10% [19,20,25,27]; another study used ≤ 10% [22]; three others used <11% [17,23,26]; two used <15% [24,29]. Moreover, some studies used different criteria to diagnose BAM: in the two case reports the response to a trial of cholestyramine was considered diagnostic for BAM [18,21]. One study used both SeHCAT test and C4 plasma concentrations > 20 ng/mL [26]; two others used C4 serum levels [28,30]; one used total fecal bile acid and primary fecal bile acid levels [31].

Most studies included patients with both LC and CC. Some included only one of the two subtypes of MC [19,20,22,26,28]. One study also incorporated patients with eosinophilic colitis [25], whereas one study did not specify the type of MC [29].

Overall, the prevalence of BAM in MC ranged from 20 to 50%, apart from four studies reporting a prevalence ranging from 9 to 18% [22,27,28,30]. Furthermore, one study reported no difference between the prevalence of MC in patients with or without BAM [29]. Some studies showed a higher prevalence of BAM associated with a specific subtype of MC, such as LC [17,23,30], or CC [27]. Furthermore, Fernández-Bañares et al. showed a greater severity of BAM in patients with CC rather than LC [17], although this difference was not significant.

In almost all studies, patients with MC and BAM were treated with cholestyramine, with a percentage of responders ranging from 70 to 80% (just one study showed a lower efficacy [22]). Bajor et al. [26] and Vijayvargiya et al. [31] treated patients with budesonide, with clinical benefits. Furthermore, the case report of Rampton et al. revealed an improvement in histologic findings of rectal biopsies after treatment with cholestyramine [18]. One study [17] showed a correlation between the severity of BAM and the number of eosinophiles in CC, but not in LC, even if a histological improvement after therapy appeared only in 3/8 LC. Another study from the same author [23] showed histological improvement in 12/23 patients after remission with cholestyramine.

### 3.2. Bile Acid Malabsorbtion and Bile Acid Diarrhea

BAD is a condition characterized by chronic watery diarrhea, associated with urgency, occasional incontinence and often abdominal pain, that affects the quality of life of patients and their work performance. Obesity seems to be a risk factor for the development of BAD [32].

Depending on the causes, BAD can be subdivided into [9]:

- Type I: secondary to ileal dysfunction, i.e., failure to reabsorb bile acids in the ileum because of resection, bypass or Crohn’s disease.
- Type II: primary idiopathic condition, in which there are decreased plasma levels of ileal fibroblast growth factor 19 (FGF19). FGF19 is produced by ileal enterocytes as a response to excess bile acids in the terminal ileum, causing a negative feedback loop on hepatic bile acids synthesis through the farnesoid X receptor (FXR) and reducing bile acids synthesis.
- Type III: gastroenterological conditions interfering with the normal bile acids reabsorption (e.g., cholecystectomy, chronic pancreatitis, small intestinal bacterial overgrowth, colitis, celiac disease, radiation-induced enteritis, diabetes mellitus).
- Type IV: excessive hepatic bile acids synthesis: observed in patients with hypertriglyceridemia or using metformin [33].

Even if BAD is usually diagnosed in adults, there is evidence documenting type II BAD in about 20% of adolescents with chronic, non-bloody diarrhea often attributed to irritable bowel syndrome [34].

The excess of bile acids arriving in the colon increases motility and electrolyte secretion, leading to diarrhea. Due to the presence of chronic diarrhea, this condition has an important negative impact on the quality of life.

The diagnosis is made with the SeHCAT test, currently representing the gold standard, where available [35]. However, since this test is not available everywhere, other diagnostic methods have been suggested, such as the serum assessment of C4 [14] or FGF19 [36]. These latter tests, unfortunately, show important circadian variability. Serum and fecal quantification of bile acids have also been evaluated: the first does not correlate with the ileal absorption, the second is somewhat difficult and tedious for patients, since it involves collection of feces for 24–48 h. Therefore, an empirical trial with cholestyramine is often used in the daily clinical practice if BAM is suspected. However, this approach is not standardized and it can yield false positive and false negative results, limiting a correct diagnosis and an effective treatment [35]. A positive SeHCAT test does not exclude other causes of organic diarrhea, e.g., Crohn’s disease with ileal inflammatory involvement, ileal resection, Whipple surgery, radio- or chemotherapy, chronic pancreatic insufficiency, cholecystectomy or Habba syndrome. Therefore, it is important to rule out possible organic disease before performing SeHCAT [16].

### 3.3. Microscopic Colitis

MC has a quite variable clinical presentation, including chronic or intermittent watery non-bloody diarrhea, often with nocturnal stools and fecal urgency. Abdominal pain, arthralgia and weight loss are less frequently reported. MC affects mainly the elderly population and risk factors include female sex, use of proton pump inhibitors, use of selective serotonin reuptake inhibitors, and active smoking. The diagnosis is histological, because colonoscopy often appears normal or shows aspecific lesions such as erosions, ulcers, or erythema. As suggested by guidelines, if MC is suspected [37], at least two biopsies of the right hemicolon and two biopsies left hemicolon should be performed and placed in two separate vials.

The treatment is based on the use of budesonide, and it appears to be effective in most patients who, unfortunately, often report a relapse after discontinuation of the therapy.

### 3.4. Association between BAM and MC

To date, there is still a poor knowledge of many physicians regarding the possibility of the simultaneous presence of BAM and MC in the same patient. Even more worrying is the fact that many patients with IBS-D of FD are often no longer investigated for these conditions when they do not respond to the usual treatments.

Nevertheless, the association between a malabsorption of bile acids in the ileum (i.e., BAM) and MC is not a rare condition (see Table 1) [17,20,38]. However, despite their prevalence, both diseases still represent an infrequent diagnosis for many gastroenterologists and in clinical practice they are often considered mutually exclusive.

The coexistence between these MC and BAM should be considered, especially when a patient with BAM or MC does not respond to the appropriate therapy because the prevalence of CC and LC in BAM is not a rare event. In our review we discovered that the prevalence of the coexistence ranged from 11% to 71% for CC and from 18% to 62% for LC, according to the different studies (Table 1).

This high variability regarding the prevalence can be explained by: different cutoffs for the SeHCAT test used to diagnose BAM from study to study; the use of other diagnostic methods rather than SeHCAT to diagnose BAM, such as dosage of C4 and FGF19. Furthermore, the population of patients with MC in the different studies was often quite heterogeneous with different percentages of CC and LC in the studied sample. All the abovementioned factors could have impacted the prevalence of the association between BAM and MC, making it difficult to understand the exact prevalence. Further studies, with standardized diagnostic methods and cutoffs and more homogeneous populations, are needed.

According to a recent review with meta-analysis, one third of patients with MC reported symptoms compatible with irritable bowel syndrome [3]. This disorder of gut–brain interaction is characterized by abdominal pain associated with defecation and/or related to changes in stool consistency or frequency of bowel movements [3]. The odds of MC were no higher in patients with IBS-D compared with other patients with diarrhea [39]. Another review from Guagnozzi et al. confirmed the same findings: almost 39.1% of MC patients (mainly with LC) also report IBS symptoms, while the prevalence of MC in patients with IBS was 7% [11].

In consideration of the high prevalence of BAM, it seems reasonable that in the diagnostic flowchart of watery diarrhea this condition should be taken into account before MC. However, since patients with the overlap of MC-BAM usually respond to budesonide, and much less frequently to cholestyramine, it is reasonable to rule out MC in patients with BAM after a therapeutic failure with cholestyramine. It is also important to stress the importance of obtaining biopsies during colonoscopy in patients with chronic diarrhea, also when the macroscopic findings are normal or not specific. The lack of a histological assessment can lead to a delayed or a missed diagnosis.

## 4. Pathophysiological Mechanisms

Although MC and BAM have a different pathophysiological background, they share some similar pathways, leading to the same clinical manifestation: watery diarrhea. Some of these pathways are discussed below.

### 4.1. Role of Farnesoid X Receptor (FXR)

**Figure 1:**
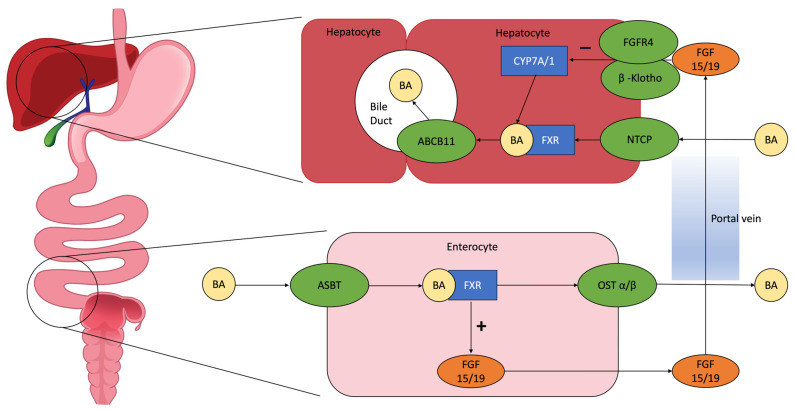
Pathophysiology of enterohepatic circulation: BAs excreted in the intestinal lumen are mainly reabsorbed in the ileum through the apical sodium-dependent bile acid transporter (ASBT) and return to the liver through the portal circulation, stimulating the farnesoid X receptor (FXR). This initiates the production of fibroblast grow factor (FGF) 15/19, which interacts in the hepatocytes with cholesterol 7 alpha-hydroxylase (CYP7A/1) and reduces BA synthesis, with a negative feedback mechanism [16]. Abbreviations: BA = bile acid; ASBT = apical sodium-dependent bile acid transporter; FXR = farnesoid X receptor; FGF 15/19 = fibroblast grow factor 15/19; OST α/β = organic soluble transporter α/β; FGFR4 = fibroblast grow factor receptor 4; CYP7A/1 = cholesterol 7 alpha-hydroxylase; NTCP = sodium-dependent uptake transporter; ABCB11 = ATP binding cassette subfamily B member 11.

Farnesoid X Receptor (FXR) is the main nuclear bile acid receptor. It is expressed in the liver and intestine, especially in the proximal colon. Binding the bile acids, it acts as a metabolic feedback sensor for their synthesis. Bile acid-dependent activation of FXR leads to two outcomes (Figure 1):

- In enterocytes, it induces the synthesis of fibroblast growth factor 19 (FGF19), which inhibits CYP7A1, the hepatic enzyme that synthetizes bile acids, producing C4;
- A decreased bile acid intestinal absorption and an increase in the expression of organic solute transporters α and β in enterocytes, in order to prevent intracellular bile acid accumulation and its excretion to the portal system [24].

Therefore, FXR-mediated mechanisms prevent the noxious effects of bile acid accumulation.

According to the literature data, patients with MC present a significantly lower expression of FXR in the colon [40]. A possible hypothesis is that colonic inflammation, through the inactivation of FXR, could render colonic epithelial cells more susceptible to the deleterious effects of bile acids, leading to the intracellular accumulation of bile acids. FXR plays an important role also in hepatic inflammation and regeneration, as well as in regulating the extent of inflammatory responses, barrier function and prevention of bacterial translocation in the intestinal tract [40]. Moreover, the lower circulating levels of FXR determine a reduction in the production of FGF19, resulting in a reduced inhibition of CYP7A1. The result is an increased production of bile acids and a reduced reuptake, possibly contributing to MC pathogenesis and symptoms [41].

### 4.2. Role of Gut Microbiota

Bile acids regulate gut bacteria growth and composition, which reciprocally regulate the circulating bile acid pool size with effects on metabolism and physiology. Bile acids are physiological detergents that solubilize dietary fats, vitamins, and xenobiotics, so they can be absorbed across the intestinal epithelium. Usually, 95% of bile acids is reabsorbed in the terminal ileum, while the remaining 5% that arrives in the colon is metabolized by the microbiota. The primary bile acids, cholic acid and chenodeoxycholic acid, are converted into deoxycholic acid and lithocholic acid, respectively. The main actions of the gut microbiota are hydroxylation and deconjugation, the latter mediated by the enzyme bile salt hydrolase (BSH). This leads to a more lipophilic composition of bile acids, which are catabolized and reabsorbed. BSH is expressed by many bacterial phyla, but its expression is higher in Bifidobacterium, Lactobacillus, Bacteroides, Enterococcus and Clostridia genera. Bile acids can determine direct damage to the bacterial membrane, preventing bacterial overgrowth, so the expression of BSH is a possible tolerance mechanism implemented by bacteria to survive bile acid exposition.

A dysbiosis could determine a reduction in BSH-producing bacteria, and consequentially a reduced deconjugation, leading to a BAM and a possible BAD. This is confirmed in experimental mice models: the absence of microbiome leads to an altered pool of bile acids in the ileum, with increased levels of bile acids, which are antagonists of FXR, leading to the lack of a negative feedback on the production of bile acids in the liver [42].

Similar mechanisms could be present also in humans. Supporting the link between dysbiosis and BAM/BAD, some studies conducted in patients with BAD found a dysbiosis as a possible cause of alteration in bile acid metabolism [43,44]. In a small number of studies, a dysbiosis was found also in MC, with a reduction in the alpha diversity of microbiota due to inflammation and an increase in the peak-to-trough ratio [45]. This ratio describes the species’ replication rate, and it is a valuable tool for investigating microbiome dynamics. In active MC, the peak-to-trough ratio is higher both in the overall microbiome and in particular for *Alistipes finegoldii* (bacteria especially involved in intestinal inflammation), compared to healthy controls and MC clinical remission. This dysbiosis, through the abovementioned mechanisms, can lead to a BAM in patients with active MC.

### 4.3. Role of Apical Sodium Dependent Bile Acid Transporter (ASBT)

The enterohepatic recirculation of bile acids involves many transport proteins (Figure 1) [46]. ASBT is the most important. ASBT is expressed on the apical membrane of enterocytes in the terminal ileum and mediates the reabsorption of bile acids into enterocytes of the terminal ileum. Bile acids bind to ileal bile acid-binding protein (IBABP) and are secreted into portal circulation by organic solute transporters α and β. These bile acids return to the liver and re-enter hepatocytes, completing the cycle of entero-hepatic circulation [45]. The importance of ASBT in bile acid recirculation can be demonstrated by a case report describing a severe BAM in patients with homozygous mutation of ASBT [47]. Due to its role in bile acid recycling, some studies have explored the possibility of inhibiting ASBT as a therapy for constipation, dyslipidemia, atherosclerosis, type 2 diabetes mellitus, non-alcoholic fatty liver disease and cholestatic liver diseases [48].

ASBT is downregulated by FXR, reducing bile acid reuptake, with the possibility of developing BAD. Studies on mice have demonstrated that in the case of experimental colitis, induced with dextran sulfate sodium, inflammation increases the level of c-fos proteins, which causes a direct inhibition of ASBT. This leads to an increase in bile acids that arrive in the colon [49]. Moreover, a study showed an activation of the human ASBT gene by direct binding of dexamethasone and budesonide, with increased reabsorption of bile acids [50]. In conclusion, ASBT is inhibited by inflammation and induced by corticosteroids; the first decreases the ileal reabsorption of bile acids, the second increases it. Therefore, type 1 BAM, which is related to ileal inflammation or resection, depends on a reduced pool of ASBT. In the same way in MC, the inflammation could induce the depletion of the ASBT of the terminal ileum. In both cases, the reduction in ASBT reduces the reuptake of bile acids in the terminal ileum and determine diarrhea.

### 4.4. Role of Takeda G-Protein-Coupled Receptor 5 (TGR5)

Another physiopathological aspect under debate is the TGR5 pathway. This is a membrane G protein-coupled bile acid receptor mainly expressed in the gastrointestinal tract. Its expression is higher in the distal ileum and colon, but it can be found also in cholangiocytes, stellate cells, sinusoidal endothelial cells and Kupffer cells of the liver. TGR5 is activated by bile acids and plays a role in insulin sensitivity, adipose tissue browning, reduction in hepatic steatosis. It is also involved in gallbladder filling and inflammation through inhibition of NFkB. TGR5 gene expression is partially regulated through FXR, indicating that FXR and TGR5 may interplay to regulate metabolism in the liver and gut [51]. TGR5 seems to have a role in [52]:

- Modulating intestinal inflammation. In a mice mode, the activation of TGR5 leads to an anti-inflammatory effect through a reduction in IL6, TNFα, INFγ and an increase in IL10.
- Maintaining intestinal barrier integrity. In a mice model, a deficit in TGR5 leads to an abnormal morphology of the colonic mucosa and increased intestinal permeability, with an altered molecular architecture of epithelial thigh junctions, increased expression and abnormal distribution of zonulin 1.
- Intestinal motility. It is expressed on enteric neurons and mediates the effects of bile acid on colonic motility. Confirming this hypothesis, a study by Alemi et al. showed that TGR5 overexpression in transgenic mice caused a more rapid colonic transit time and increased frequency of defecation [53].

Therefore, a possible dysregulation of TGR5 induced by bile acids could play an etiopathogenetic role in inflammatory diseases of the colon [34], such as MC.

## 5. Conclusions

The clinical management of IBS-D and FD may be complex and uncertain [1,5,54]. Guidelines point towards a positive diagnostic approach [47]. However, in the presence of symptoms non-responsive to conventional therapies, in the absence of alarm symptoms, organic diseases (i.e., BAM and MC) must be ruled out. BAM is not a rare disease, and its prevalence is higher than MC in patients with chronic watery diarrhea. For this reason, in non-responder patients it is mandatory to first rule out BAM by performing a SeHCAT test, where available or evaluating the serum levels of C4 and/or FGF19. In the meantime, as highlighted by pathophysiological mechanisms, MC patients could display a secondary BAM. For this reason, in patients with a proven BAM who are unresponsive to bile acid sequestrants, a colonoscopy with multiple biopsies may help to address the problem.

It is important to perform multiple biopsies during colonoscopy while evaluating patients with chronic watery diarrhea, even when the colonic mucosa appears to be macroscopically normal, since this is the only way of reliably diagnosing MC.

Further research should focus on:

- Exploring the exact prevalence of the association of BAM and MC in chronic watery diarrhea or in patients labelled as “functional” but refractory to conventional therapy;
- Clarifying the role of the most debated molecular actors (e.g., TGR5) involved in BAM pathophysiology and its relationships with MC.
- Understanding the relationship between BAM and MC in symptom generation, when both coexist in the same patients;
- Discovering the correct treatment strategy in these patients through randomized controlled clinical trials.

## Figures and Tables

**Table 1:** Studies evaluating the possible association between MC and BAM.

	Type of Study	Age and Gender	Symptoms	Criteria to Define BAM	Type of MC	Prevalence of BAM in MC	Associated Treatments	Response to Therapy	Histology after Therapy
Rampton (UK, 1987) [18]	Case report	32 years old; F	10 daily bowel movements, occasionally night evacuation, predefecatory lower abdominal pain.	Response to a trial with cholestyramine. After a week off cholestyramine SeHCAT test showed an increased fractional turnover rate over the gallbladder but a normal daily absorptive efficiency.	NA	1/1	Loperamide	No response to oral sulfasalazine, mebeverine, and metronidazole, or topical corticosteroids. Response to cholestyramine (4 g, 3 bowel movements/day of normal feces).	Rectal biopsies taken after cholestyramine intake for three months were normal.
Bohr (Sweden, 1996) [19]	Retrospective observational study	Median age 55 (18–87) years old; 142/163 (87%) F, 21/163 (13%) M	Chronic diarrhea	SeHCAT test with a retention <10% on day 7 was considered abnormal.	163 CC	10/26 CC that responded to cholestyramine had abnormal SeHCAT test	Sulphasalazine (108/163); mesalazine (16/163); olsalazine (15/163); prednisolone (39/163); budesonide (2/163); metronidazole (44/163); erythromycin (15/163); penicillin (8/163); mepacrine (19/163); loperamide (69/163)	NA	NA
Ung (Sweden, 2000) [20]	Uncontrolled clinical study	NA, 22/27 (81%) F, 5/28 (19%) M	Chronic diarrhea	SeHCAT test with a retention <10% on day 7 was considered abnormal.	27 CC	Abnormal SeHCAT test (<10%) in 12/27 (44%) of CC patients.	4/27 (15%) non responders to bile acids sequestrants were prescribed sulphasalazine 1 g 2 daily was prescribed for 2 months. 1/27 (4%) non responder to sulphasalazine too, was prescribed metronidazole 0.4 g 3 times daily for 2 weeks followed by 0.4 g 2 daily for 6 months	- 24 patients were prescribed cholestyramine, 2 had colestipol prescribed. - Improvement with bile acid sequestrants in 23/27 (85%) patients.	NA
Fernández-Bañares (Spain, 2001) [17]	Uncontrolled clinical study	Median age 60.7 ± 2.2, 41/51 (80%) F, 10/51 (20%) M for MC. Median age 55.6 ± 3.2, 3/26 (12%) F, 23/26 (88%) M for CC. Median age 65.1 ± 2.7, 18/25 (72%) F, 7/25 (28%) M for LC	- Chronic or recurrent watery diarrhea of atleast one month’s duration. - No significant differences in fecal frequency and consistency, and in percentage of patients with urgency between microscopic colitis patients with or without BAM.	- SeHCAT test with a retention <11% on day 7 - Values lower than 5% on day 7 were considered as severe BAM.	26/51 (51%) CC 25/51 (49%) LC	- 22/51 (43%) of patients with MC had BAM - 7/26 (27%) CC and 15/25 (60%) LC (*p* < 0.025) had BAM - Excluding 7 patients with previous cholecystectomy (2 CC and 5 LC), 34% of MC had BAM: 5/24 (21%) with CC and 10/20 (50%) with LC (*p* < 0.059) - A higher, but not significant, proportion of patients with severe BAM in CC than in LC (71.4% vs 33.3%; *p* < 0.17)	Before prescription of cholestyramine: - 9 patients were taking mesalazine (500 mg three times a day) - 2 patients were taking mesalazine plus oral prednisone (1 mg/kg/day)	- 19/22 (86%) of patients with MC and BAM responded to cholestyramine with decrease in daily stool number (before therapy: 5 stools/day; after: 1 stool/day; *p* < 0.0001), increase in fecal consistency (before therapy: 22 liquid/semiliquid; after: 3 liquid/semiliquid, 19 formed/semiformed; *p* < 0.0005) and decrease of urgency that disappeared in all 12 patients with this symptom. - Patients with CC required significantly higher doses of the drug than those with LC (median, 8 g/day vs. median, 4 g/day; *p* < 0.0014). Median time to achieve clinical remission was five days - All 19 patients on cholestyramine had no relapse of diarrhea after a mean follow up of 24.9 ± 2.9 months - 7 patients stopped cholestyramine after 10.2 ± 2.5 months without clinical relapse in the following 6 months.	- In patients with CC and BAM, there was a significant correlation between the severity of BAM and the number of IEL (*p* < 0.042). - No correlation with thickness of subepithelial collagen layer in CC with BAM nor between the severity of BAM and the number of IEL in LC. After follow-up of 21.5 months 8/19 patients with MC and BAM clinically responsive to cholestyramine went to colonoscopy with biopsies. Histological improvement was observed in 3/8 LC. No patient with CC improved.
Gurbuz (Turkey, 2001) [21]	Case report	44-yr-old; M	Cholecystectomy for cholelithiasis. On the third postoperative day he had high volume watery diarrhea with loss of approximately 500 mL/24 h and a frequency of three times per day	Response to cholestyramine 12 g/die	LC	1/1	NA	Stool volume and frequency progressively decreased; consistency improved. Symptoms relapsed after drug withdrawal.	NA
Ung (Sweden, 2002) [22]	Abstract of uncontrolled clinical study	NA	Chronic diarrhea	SeHCAT <\=10%	23 LC	2/23 (9%) with LC had abnormal SeHCAT (≤10%). No correlation between the SeHCAT values and the degree of colonic inflammation	NA	6/13 (46%) responded to bile acid sequestrants	NA
Fernández-Bañares (Spain, 2003) [23]	Uncontrolled clinical study	Median age 57.0 ± 2.3 in CC, 65.5 ± 2.3 in LC Gender 5/37 (14%) M and 32/37 (86%) F in CC; 11/44 (25%) M and 33/44 (75%) F	Chronic or recurrent watery diarrhea of at least 1-month duration.	SeHCAT test <11% on day 7	37/81 (46%) with CC 44/81 (54%) with LC	In 8/26 (31%) of patients with CC and in 16/26 (62%) of patients with LC BAM was diagnosed	13/37 (35%) patients with CC and 8/44 (18%) with LC were taking NSAIDs at diagnosis. 16% and 20% of patients with CC and LC, respectively, were on antidepressants.Patients with MC and BAM who failed cholestyramine were treated with mesalazine. Those failing mesalazine were treated with prednisone, and in the last years, with budesonide with controlled ileal release.	- Cholestyramine, at a mean dose of 8 g/day (range 2–12 g/day), was more effective in LC than in CC (*p* < 0.06). However, there were no differences in patients with concomitant BAM, being as effective in patients with CC (75% resolution of diarrhea) as in those with LC (86% resolution of diarrhea)	- 23 patients (10 with CC and 13 with LC) underwent again colonic biopsies after a median follow-up of 24 months. - 18/23 patients were on maintenance treatment (10 with cholestyramine, 8 with mesalazine,) Histomorphological improvement was observed in 12/23 patients in clinical remission.
Wildt (Denmark, 2003) [24]	Uncontrolled clinical study	SeHCAT <15% was found in 74/133 (56%) patients with chronic diarrhea; 46/74 (62%) F, 28/74 (38%) M. No data for MC group	Chronic diarrhea defined as change in stool frequency and/or consistency for more than 3 weeks	SeHCAT test <15% on day 7	NA	Of 74 patients with SeHCAT <15%: 12/74 (16%) type 1 BAM, 24/74 (33%) type 2 BAM, 38/74 (51%) type 3 BAM 9/23 (39%) of patients with MC showed SeHCAT <15%	NA	Criteria for remission: reduction of 25% of frequency, or file data reporting excellent or moderate response to treatment - Treatment with cholestyramine led to a response in 70% of patient - Best treatment responses were seen in 82% of patients with type 2 BAM versus 65% in patients with types 1 and 3 BAM. Treatment response was 74% in patients with severe BAM vs. 65% in patients with moderate and mild BAM	NA
Müller (Sweden, 2004) [25]	Prospective observational study	In 158 patients with non-bloody diarrhea mean age was 46 (range 16–84); 103/158 (65%) F, 55/158 (35%) M	Three or more loose stools daily and/or a substantial increase in stool frequency and/or fluidity for more than 4 weeks	SeHCAT test <10% on day 7	34 patients: 8 LC, 12 CC. In the study 14 eosinophilic colitis were also included	8/34 (26%)	NA	NA	NA
Bajor (Sweden, 2006) [26]	Uncontrolled clinical study	The median age in the collagenous group was 59 years (53 and 70 years) and the gender distribution was 22/25 (88%) F, 3/25 (12%) M. The median age in the control group was 45 years (25 and 47.5) and the gender distribution was 21/29 (72%) F and 8/29 (28%) M	Chronic diarrhea	SeHCAT test <11% on day 7, C4 plasma concentrations over 20 ng/mL were also evaluated	25 CC	6/25 (24%)	Before initiation of the treatment with budesonide there was a wash out period of at least 2 weeks of antidiarrheals and drugs with a potential effect on BA metabolism, including corticosteroids. These drugs were not allowed during the study period	After treatment with budesonide 9 mg daily for 8 weeks median 75SeHCAT retention increased from 18% to 35% (*p* < 0.001) (6 patients (24%) had an abnormal value initially). The C4 values decreased significantly (from 36 to 23 ng/mL, *p* = 0.04). The 29 healthy controls 75SeHCAT values were 38% (29% and 48%). The difference between the collagenous colitis group and healthy controls (*p* < 0.0001) disappeared during the treatment (*p* = 0.26)	NA
Bjørnbak (Denmark, 2011) [27]	Retrospective observational study	The median age was (years) 65 CC, 63 LC, 62 MCi. The gender distribution was 200/270 (74%) F and 79/270 (26%) M in CC, 108/168 (64%) F and 60/168 (36%) M in LC, 83/101(82%) F and 18/101 (18%) in MCi	Watery diarrhea with often associated nightly evacuations, urgency, abdominal pain and sometimes loss of weight	SeHCAT test <10% on day 7	539 (168 with LC, 270 with CC, 101 MCi)	70/270 CC (26%), 30/168 LC (18%), 64/101 MCi (63%)	Concomitant therapies at diagnosis:NSAID use was 54/270 (20%) in CC, 20/168 (12%) in LC, 26/101 (26%) in MCi. Salicylic acid use was 81/270 (30%) in CC, 44/168 (26%) in LC, 27/101 (27%) in MCi Proton pump inhibitor use was 89/270 (33%) in CC, 37/168 (22%) in LC, 35/101 (35%) in MCi	Efficacy of different therapies: Psyllium 36⁄86 (42%) CC, 14⁄40 (35%) LC, 7⁄15 (47%) MCi. Psyllium + Calcium 20⁄50 (40%) CC, 5⁄13 (38%) LC, 7⁄15 (47%) MCi Cholestyramine 39⁄95 (41%) CC, 23⁄43 (52%) LC, 22⁄29 (76%) MCi Loperamide 30⁄49 (61%) CC, 11⁄22 (50%) LC, 5⁄6 (83%) MCi Antibiotics 1⁄13 (6%) CC, 2⁄10 (20%) LC, 3⁄10 (30%) MCi 5-ASA 4⁄28 (14%) CC, 1⁄9 (11%) LC, 1⁄6 (17%) MCi Budesonide 135⁄161 (84%) CC, 86⁄98 (88%) LC, 26⁄31 (84%) Azathioprine 7⁄22 (32%) CC, 1⁄10 (10%) LC, 0⁄3 (0%) MCi	NA
Brydon (UK, 2011) [28]	Prospective observational study	NA	Chronic diarrhoea	7-alpha-hydroxy-4-cholesten-3-one serum levels were used to diagnose BAM	6 CC	1/6 CC (17%)	NA	NA	NA
Appleby (UK, 2017) [29]	Retrospective observational study	SeHCAT >15%: 275 patients, mean age (years) 48.7, gender distribution 176/275 (64%) F, 99/275 (36%) M SeHCAT <15%: 303 patients, mean age (years) 52.1, gender distribution 184/303 (61%) F, 119/303 (39%) M	Chronic diarrhea	SeHCAT test <15% on day 7	NA	328 had a completed colonoscopy, of which 172 had a SeHCAT <15% and 156 had a SeHCAT >15%. Two hundred patients had colonic biopsies, of them: - SeHCAT >15% in 101 patients; 4/101 (4%) had microscopic colitis. - SeHCAT <15% in 99 patients; 5/99 (5%) had microscopic colitis.	NA	NA	NA
Davie (UK, 2020) [30]	Retrospective observational study	NA	Chronic diarrhea	Values of 7-alpha-hydroxy-4-cholesten-3-one measured >22 ng/mL were diagnostic for BAM	140/646 CC had 7αC test 20/204 LC had 7αC test	- 16/140 (11%) CC had BAM - 5/20 (25%) LC had BAM	NA	NA	NA
Vijayvargiya (USA, 2022) [31]	Uncontrolled clinical study	Mean age (years) 66 for CC, 58 for LC. Gender distribution: 27/34 (79%) F and 7/34 (21%) M in CC; 29/32 (91%) F and 3/32 (9%) M in LC.	Chronic diarrhea	BAM was defined as elevated total fecal bile acids (>2337 μmol total bile acids/48 h) or elevated primary faecal bile acids (>10% primary bile acids or >4% primary bile acids + >1000 μmol total bile acids/48 h).	66 MC (34 CC, 32 LC)	BAM was found in approximately 50% of patients with MC	NA	All patients with MC (with or without BAM) were treated with budesonide. 17/45 (38%) of MC did not improve, 6/17 (35%) had bile acid diarrhea, and 2/6 (33%) improved with BAs.	NA

Abbreviations: CC = collagenous colitis; LC = lymphocytic colitis; BAM = bile acid malabsorption, IEL = intraepithelial lymphocytes; BAs = bile acid sequestrant.

## Data Availability

Data sharing not applicable. No new data were created or analyzed in this study. Data sharing is not applicable to this article.
